# Leveling the Research Playing Field: Decolonizing Global Health Research Through Web-Based Platforms

**DOI:** 10.2196/46897

**Published:** 2023-10-31

**Authors:** Rajeev Seth, Baldeep K Dhaliwal, Emily Miller, Tyler Best, Alexis Sullivan, Betty Thankachen, Yawar Qaiyum, Anita Shet

**Affiliations:** 1 Bal Umang Drishya Sanstha New Delhi India; 2 Department of International Health Johns Hopkins Bloomberg School of Public Health Baltimore, MD United States; 3 Center for Communication Programs Johns Hopkins Bloomberg School of Public Health Baltimore, MD United States; 4 International Vaccine Access Center Johns Hopkins Bloomberg School of Public Health Baltimore, MD United States

**Keywords:** decolonization, vaccination, community, community engagement, health equity, health research, online, online platform, web-based platform, systemic barrier, diversity, marginalized, promote, equity, research

## Abstract

Global health research has traditionally been rooted in colonialism, with some investigators in high-income countries leading and managing research and investigators in low- and middle-income countries serving as implementing partners. The Community Health Worker-Led Intervention for Vaccine Information and Confidence (CIVIC) Project, conducted in India and led jointly by India- and US-based investigators, leveraged web-based platforms to facilitate a more horizontal, inclusive, and balanced approach to partnerships between researchers and the community. Using web-based platforms to conduct research was found to be an effective strategy to engage researchers at all levels and combat systemic barriers associated with in-person activities such as power, economic, social, and gender dynamics. Connecting online for research meetings created a more equitable environment for community members to engage meaningfully with research. Further, by conducting research through web-based platforms, we found that we were able to strengthen the diversity of participants, provide a space for more marginalized groups to speak up, and minimize logistical barriers to attendance. Harnessing web-based approaches in research provides a pathway toward opportunities to promote equity and contribute to the decolonization of global health spaces.

## Introduction

Research collaborations have historically relied on in-person gatherings to implement research and interventions. In global health specifically, in-person gatherings have perpetuated cycles of colonialism, as research collaborations have traditionally functioned as partners in high-income countries advising investigators in low- and middle-income countries (LMICs). In the initial phases of the COVID-19 pandemic, in-person gatherings were difficult to sustain, triggering discussions on current and future formats of research and how these formats can contribute to decolonization.

The Community Health Worker-Led Intervention for Vaccine Information and Confidence (CIVIC) Project, conducted in Mewat, India, incorporated the use of web-based platforms to lead to a more horizontal and inclusive approach to partnerships between researchers and the community [1]. In bringing together a diverse range of perspectives, our team aimed to conduct effective research and facilitate long-lasting, sustainable changes in community health, led by in-country investigators and community leaders. In this project, we also developed a community accountability board (CAB) of local influential leaders to ensure that the learnings from this project would last beyond the period of the grant. The onset of the COVID-19 pandemic, followed by national lockdowns and travel limitations, led to innovations in bringing together in-country stakeholders and frontline health workers to lead health initiatives [2]. This viewpoint highlights a pathway toward an approach to contribute to the decolonization of global health.

## Engaging Stakeholders in Community Research

Stakeholders, defined as any person or entity who is impacted by the process or organization of a research plan [3], can facilitate the creation of targeted interventions using unique perspectives [4] and can ensure that interventions are most beneficial to community members in the long term [5]. Stakeholder engagement is particularly essential as their involvement in every phase of research facilitates the following: their buy-in may lead to more relevant research questions, their involvement can improve transparency about the research process, and their engagement can accelerate the transfer of research findings into practice [6]. Although the role of stakeholders in health research in high-income countries is heavily documented, there is limited information on how to effectively engage stakeholders and understand their role in the rural South Asian context [7].

In the CIVIC Project specifically, our unique approach was to recognize community members as subject matter experts and ensure their full participation in the research [1]. To do so, our team leveraged web-based platforms to engage in community-based participatory research to involve the community from conception to implementation of an intervention. We engaged 10 community leaders, ranging from teachers to religious leaders, to form a CAB. We used web-based platforms to further complete baseline data collection with community health workers, CAB members, and caregivers of children to understand vaccination barriers and facilitators. After identifying barriers and facilitators, we conducted 2 web-based human-centered design workshops to develop a tailored intervention to improve vaccine uptake. While implementing this intervention, we used web-based platforms to meet monthly with the CAB over 4 months. Through these meetings, the CAB guided our team on where to iteratively refine the intervention. Lastly, we collected postintervention data from community health workers, CAB members, and caregivers of children through web-based platforms to assess perceptions of the intervention, as well as perceptions of its impact on vaccine uptake.

## The Case for Decolonizing Global Health Research

Decolonizing global health involves removing all forms of supremacy in all spaces of global health practice and research, including within countries, between countries, and at the global level [8]. Colonization was historically justified by delusions of supremacy; this included an epistemic supremacy that individuals in LMICs could not be producers of knowledge, and they contributed to “epistemicide” or the destruction of knowledge systems [8,9]. This has resulted in the assumption today that communities in LMICs do not have, or are incapable of, generating solutions to their own challenges. Colonization has further contributed to a refusal to learn from people who are local experts and a failure to understand that there are many ways to approach public health issues [10]. However, participatory research and embracing indigenous ways of knowing have repeatedly produced more in-depth knowledge than what would be possible through an outsider’s approach [11]. Additionally, computational research has also demonstrated how colonialism, and subsequent decolonization, have been incorporated into research design approaches [12,13].

Current research systems require reform and the creation of forums, so that global health can embrace the direct expertise of the very people it intends to serve and address their needs. In these forums, it should be local community members sharing their needs and trusted community leaders developing tailored solutions. Global health researchers from high-income settings, both from within countries and in north-south collaborations should serve as advocates for the communities in which they are conducting research instead of dictating research. The aim should be to create a space for their voices, lived experiences, and expertise and to push community priorities to the forefront of discussions.

## How Web-Based Platforms Can Level the Playing Field for Stakeholders

One of the most important approaches employed in our research to facilitate meetings, complete data collection, implement interventions, and disseminate findings to the community was to use web-based platforms to engage stakeholders. Using web-based platforms provided an opportunity to combat systemic barriers that in-person activities often facilitate. Traditionally, in-person meetings have the potential to be exclusionary in terms of gender, race, and background, particularly for topics related to science, medicine, and engineering [14]. Professional hierarchies often hinder more junior stakeholders from speaking out in the physical presence of more senior stakeholders. In-person meetings in rural India have been known to reinforce existing structural barriers and power dynamics by limiting participation from women with disproportionate family care responsibilities, individuals with greater financial obstacles, or people with disabilities [15]. Additionally, discrimination and uneven power dynamics also often manifest in in-person events, particularly in the male-dominated rural South Asian context. Creating web-based opportunities to engage with community stakeholders worked to lift these barriers to participation in the CIVIC Project.

## Lessons Learned From Using Web-Based Platforms

Web-based platforms for meetings and research can be essential tools to contribute to the decolonization of global health. Using such forms of engagement in the CIVIC Project brought together a high number of women who were typically unable to participate in these types of community engagement activities due to societal expectations or logistical household barriers. Web-based platforms further allowed stakeholders to engage virtually while still completing domestic responsibilities. Additionally, the web-based platform allowed more people, particularly young adults and women, to have the opportunity to speak. A striking lesson from our project was that using web-based platforms allowed our team to facilitate trust-building exercises and engagement with religious leaders in Mewat [16]. In addition, different ethnic and religious backgrounds in India have distinct and often hierarchical dynamics, but these can be unified when using web-based platforms for research training and communication.

Through web-based platforms, researchers are more able to overcome economic and travel-related barriers, which contribute to poor participation from institutions and countries with limited resources, women who bear more social costs to attending in-person meetings, and early career practitioners [14,15]. By conducting the CIVIC Project through web-based platforms, we found that we were able to strengthen the diversity of participants, provide a space for more marginalized groups to speak up, and minimize logistical barriers to attendance. An important lesson learned from using web-based platforms is that leveraging local knowledge through these methods enriches the exploratory nature of the research and increases the chances of successful implementation.

## Challenges and Opportunities Associated With Web-Based Platforms

Recognizing challenges in the use of web-based platforms in rural settings can provide opportunities for their widespread use to decolonize global health research. In 2016, reports indicated that nearly 90% of India’s population was considered digitally illiterate [17], creating the perception that web-based platforms may be less successful in this setting. A deeper look illustrates the obsolescence of this information as it is clear that since 2019, rural India has seen a 45% increase in active internet users, increasing their ability to connect more broadly [18]. With the recent market permeation of inexpensive electronics, smartphone ownership in India is near ubiquitous and cuts across socioeconomic strata [19]. Throughout the pandemic, digital platforms have been extensively used, further encouraging the use and familiarity of such platforms [19]. To effectively engage rural and low-income stakeholders, it is critical to remove assumptions about the capabilities of community members that facilitate a myopic and limiting approach to community engagement, thus reducing its potential impact.

The lessons on decolonization presented here are limited by small numbers, uncertain generalizability, and potential concerns around the long-term sustainability associated with short-term projects. Notwithstanding these limitations, this study provides a window into how digital approaches can facilitate local capacity, drive in-country leadership, and return power to communities.

## Conclusion

Diversifying our approaches to strengthen advocacy and scale up connectivity for these populations is an essential place to begin. Although using web-based platforms to facilitate meetings and workshops enables communities to have a larger voice in the planning of research activities and the creation and implementation of interventions, researchers must still grapple with doing more to ensure equity, particularly in populations without internet access. To further strengthen equity, there is a need to recognize the power of digital strategies in facilitating the meaningful participation of underrepresented groups in all aspects of research. As we continue to navigate COVID-19 and the multisectoral engagement that it facilitates, it is essential to harness novel technology and approaches to promote equity and contribute to the decolonization of global health spaces that have been historically dominated by external voices.

## Abbreviations

CAB: community accountability board
CIVIC: Community Health Worker-Led Intervention for Vaccine Information and Confidence
LMIC: low- and middle-income country
